# Supplementary figures and images for: Diversity and structure of the microbial community in rhizosphere soil of Fritillaria ussuriensis at different health levels
Source: PeerJ. 2022 Jan 25;10:e12778. doi: 10.7717/peerj.12778 (PMC8796711; doi:10.7717/peerj.12778)

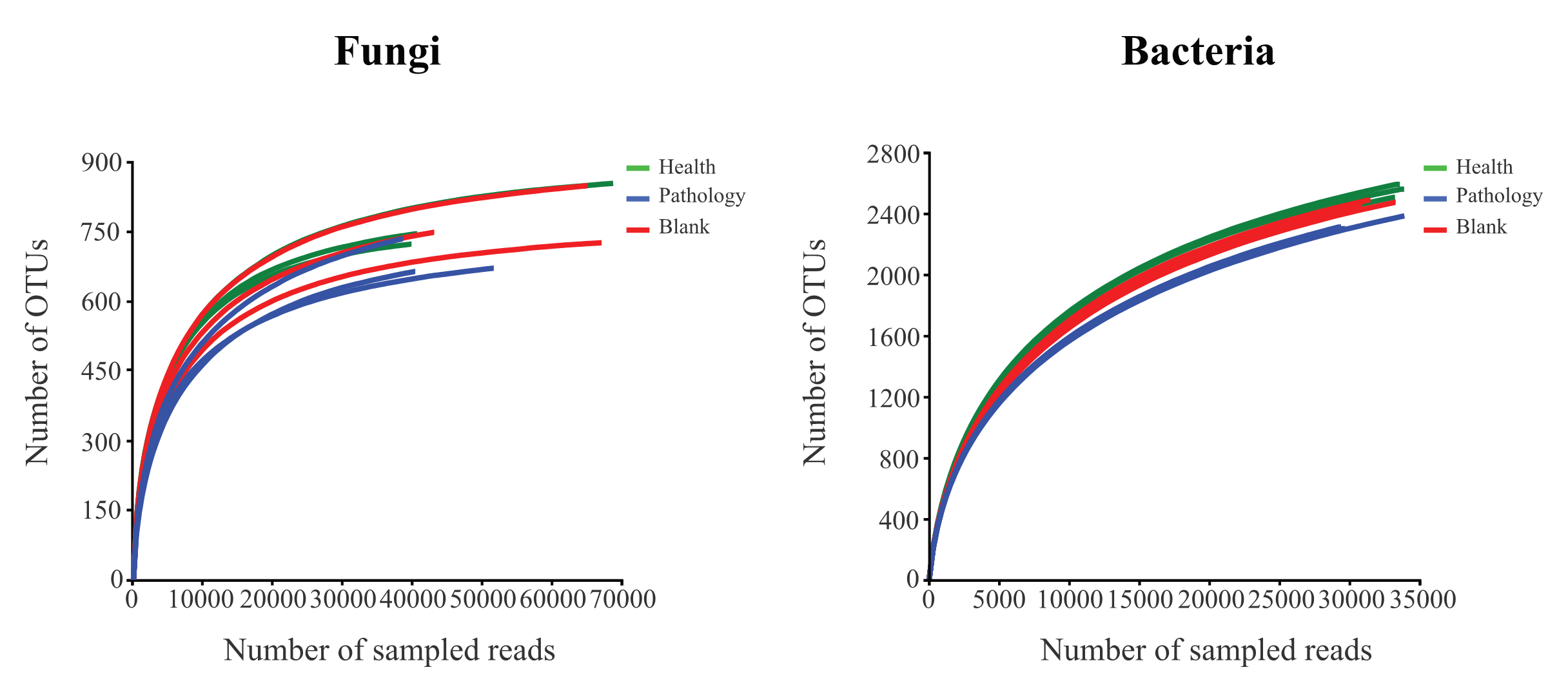

Supplement: Supplemental Information 3 [file peerj-10-12778-s003.png]
